# Supplementary material for: Sensory Integration Disorders in Patients with Multiple Sclerosis
Source: J Clin Med. 2022 Sep 1;11(17):5183. doi: 10.3390/jcm11175183 (PMC9457514; doi:10.3390/jcm11175183)
Supplement: Supplementary file 1 [file jcm-11-05183-s001.zip › jcm-1858623-supplementary.pdf]

# General: Symptoms of SPD in Adults

Here is a very basic, traditional checklist for adults with SPD. The list may seem very long, but it is a condensed version that covers the full range of symptoms adults with Sensory Processing Disorder can have. All checklists are made to print well, so feel free to print this out and mark how often and/or severely you experience each of the following symptoms on a scale of 0 (never) to 4 (always/severe), or use P, if it was previously an issue that no longer presents itself in your daily life.

---

## Sensory Modulation

**General Modulation**

_______ have unusual eating habits (strong preferences, eating at odd times, etc.)

_______ have unusual sleeping habits or sleep schedule

_______ have great difficulty with transitions, be they major life changes or small everyday stuff (one activity to another, going from inside to outdoors, etc.)

_______ become engrossed in one single activity for a long time and seem to tune out everything else

_______ spend hours at a time on fantasy or video games and activities

_______ very high or very low energy level

_______ are resistant to change in your daily life and environment

_______ may have turned to forms of substance abuse

_______ drink excessive amounts of coffee or caffeinated beverages

**Over-Responsiveness**

_______ bothered by clothes; certain materials, tags, seams, pantyhose, ties, belts, turtlenecks. May have to wear shorts, skirts, or pants exclusively, etc.

_______ bothered by "light touch"; someone lightly touching/rubbing your hand, face, leg or back

_______ excessively ticklish

_______ distressed by others touching you; would rather be the "toucher" than the "touchee". This may lead to difficulty "snuggling" with your partner

_______ very sensitive to pain, especially as compared to others

_______ dislike the feeling of showers or getting splashed

_______ difficulty going to the beach; the sand blowing on your skin or getting on your body

_______ avoid touching anything "messy"; if you do, you have to go wash your hands right away and/or only touch it with your fingertips

_______ can not wear new or "stiff" clothes that have not been washed or soaked in fabric softener

________ hate to be barefoot or hate to wear shoes and/or socks

________ frequently get car sick, air sick, motion sick

________ difficulty riding on elevators, escalators, or moving sidewalks

________ avoid amusement park rides that drop, climb, spin, or go upside down

________ difficulty eating foods with mixed textures, or one particular texture

________ prefer very bland foods, dislike anything spicy

________ become nauseated or gag from certain cooking, cleaning, perfume, public restroom, or bodily odours

________ become overstimulated or over-aroused when people come to the house or when in crowded places

________ overly exited/aroused in group settings

________ avoid crowds and hide or disappear when guests come over

________ sensitive to noises that other people do not seem bothered by (clocks, refrigerators, fans, people talking, blenders, vacuum cleaners, animals, outdoor construction, etc.)

________ easily distracted by auditory or visual stimuli

________ can not attend certain public events or places due to excessive noise

________ overreact to loud noises, like sirens

________ can't sleep if room isn't completely dark

________ fearful of heights

________ bothered by hands or face being dirty

**Under-Responsiveness**

________ may fail to recognize stimuli that most would find alerting or strong

________ unable to identify foods that have gone bad by smell

________ difficulty being able to smell dangerous smells, i.e., smoke, noxious/hazardous solvents, or if something is burning in the stove or oven

________ don't seem to notice pain; get shots/cuts/bruises and hardly feel a thing

________ lethargic, hard to get going, appear "lazy" and unmotivated

________ don't seem to notice if your hands or face are dirty

________ don't seem to get dizzy

________ have a hard time feeling parts of your body, as though they aren't there

________ difficulty waking up in the morning (may not even notice alarm clock)

**Sensory Seeking**

________ have to fidget and "fiddle" with things all the time; change in your pocket, your keys, a pen/pencil, paper clip, rubber band, anything within reach

________ often touching and twisting your own hair

________ are a thrill seeker; love fast and/or dangerous rides, leisure activities, and sports

________ seek out fast, spinning, and/or upside-down carnival rides

________ will often rock or sway body back and forth while seated or standing still

________ frequently tip chair on back two legs

________ restless when sitting through a lecture, presentation, or movie

________ constantly chew on pens and pencils, chew gum, or smoke cigarettes

________ prefer foods with very strong tastes and flavours

________ constantly bite nails or fingers

________ bite lips or inside of cheeks

________ frequently shake your leg while sitting or falling asleep

________ love to sleep with multiple or heavy blankets on top of you

________ seek out crashing and "squishing" activities

________ crack knuckles often

________ love crunchy foods (popcorn, carrots, chips, nuts, pretzels, etc.)

________ have an "endless" supply of air fresheners, scented candles, odour masking sprays, etc.

________ identify objects by smell, have to smell everything, judge whether you like something or someone by smell

________ great difficulty settling body down for sleep

________ love to touch and be touched, have to touch everything

## Sensory Discrimination

________ can't identify objects by feel if your eyes are closed

________ difficulty finding things in your purse or pocket without looking

________ difficulty heating food to the correct temperature, feeling if it is too hot or too cold

________ difficulty locating items in a cupboard, drawer, in your closet, or on a grocery shelf

________ difficulty with recognizing/interpreting/following traffic signs

________ difficulty judging distances about where your car is in relation to other cars, in parking spaces, or near a curb (fail miserably at parallel parking!!)

________ difficulty merging into oncoming traffic on road, rotary, or highway

________ get disoriented and/or lost easily in stores, buildings, hiking, etc.

________ difficulty concentrating on or watching a movie/tv show when there is background noise or distractions

________ difficulty remembering or understanding what people are telling you

________ difficulty following directions if given two or three at one time

________ can not complete concentrated tasks if noises present

________ talks too loud or too soft

________ difficulty licking an ice cream cone neatly

________ difficulty with speech and annunciation

________ bump into things frequently

________ often push too hard on objects, accidentally breaking them

________ difficulty judging how much pressure to apply when doing tasks or picking something up

________ difficulty identifying which key on your ring belongs to what

________ often reverse numbers and letters or process them backwards

________ difficulty telling time on an analogue clock

________ difficulty reading and understanding a map, bus schedule, directions

________ difficulty organizing and grouping things by categories, similarities, and/or differences

________ can't reading text on computer screens

________ difficulty distinguishing different tastes and/or flavours of food and/or drink items

________ difficulty lining up numbers correctly for math problems and/or balancing a checkbook

## Sensory-Based Motor Abilities

________ have difficulty learning to ride a bike or other moving equipment

________ clumsy, uncoordinated, and accident prone

________ difficulty walking on uneven surfaces

________ difficulty with fine motor tasks such as buttoning, zipping, tying, knitting, sewing, playing games with small parts, closing zip lock bags

________ confuse your right and left sides

________ prefer sedentary tasks, avoiding sports or physical activities

________ difficulty with handwriting; hard to read, write slowly, get wrist cramps

________ frequently bump into people and things

________ easily fatigued with physical tasks

________ frequently miss when putting objects on a table

________ messy eater, difficulty using eating utensils, spilling and dropping food frequently

________ often knock drinks or other things over when reaching for them

________ frequently drop items

________ feel the need to talk yourself through tasks

________ often hum or talk to self while concentrating on a task

________ significant difficulty learning to tie things (shoes, bags, etc.)

________ difficulty with motor tasks requiring several steps

________ difficulty learning new motor tasks (a new dance, sport or exercise activity, how to drive, etc.)

________ lose balance frequently, maybe even when standing still

________ significant difficulty learning to type without looking at the keyboard

## Social and Emotional

________ dislike changes in plans or routines, needing structure

________ often described as "stubborn", "defiant", or "uncooperative"

________ are very emotional and sensitive, may also be prone to crying

________ can't seem to finish anything

________ have difficulty making decisions

________ are seen as rigid and controlling

________ prefer solitary activities rather than group participation

________ are often impatient and/or impulsive

________ don't always register or understand social cues and non verbal language

________ difficulty with authority figures

________ trouble relating to and socializing with peers and colleagues

________ have difficulty accepting defeat or forgiving yourself

________ frequently get strong feelings of anger or rage

________ easily frustrated

________ needs sameness and routines; needs to know what to expect

________ get frequent panic or anxiety attacks

_______ have many fears and/or phobias

_______ OCD-type qualities; can't let foods touch each other on your plate, have to wear clothes a certain way, or other obsessions and compulsions.

_______ are easily distractible and often unorganized

_______ hate surprises

_______ difficulty seeking out and maintaining relationships

_______ avoid eye contact

### Internal Regulation

_______ difficulty falling asleep or getting on a sleep schedule

_______ heart rate issues, including: unnecessary speeding, not slowing down when at rest, or not speeding up for tasks that require a higher heart rate

_______ respiration too fast or slow for the appropriate state of arousal

_______ over or under sensitive to bowel and bladder sensations

_______ over or under sensitive to the sensations of hunger and thirst

_______ irregular, inconsistent bowel, bladder and appetite sensations

_______ difficulty with temperature regulation of body

---

The above checklist was edited by Dan Travis and reposted from sensory-processing-disorder.com with the original author's permission.
